# *Aristolochia vallisicola* (Aristolochiaceae), a new species from Peninsular Malaysia

**DOI:** 10.3897/phytokeys.14.3354

**Published:** 2012-07-26

**Authors:** Tze Leong Yao

**Affiliations:** 1 Forest Research Institute Malaysia, 52109 Kepong, Selangor, Malaysia

**Keywords:** Aristolochiaceae, *Aristolochia*, Peninsular Malaysia

## Abstract

A new species in the genus *Aristolochia* (Aristolochiaceae), *Aristolochia vallisicola* T.L.Yao, from Peninsular Malaysia is described and illustrated. Among all Peninsular Malaysian *Aristolochia*,itis the only species with a pinnately veined lamina and a disc-liked perianth limb. A distribution map is provided and its conservation status is assessed as Least Concern.

## Introduction

*Aristolochia*, the largest genus in the family, consists of about 400 species. It is widely distributed throughout tropics and subtropics, but also in the warm temperate regions. Hou (1984) recognised 28 species in Malesia, 5 of which occur in Peninsular Malaysia while none of them is endemic.

The new species presented here was first collected by a Forest Guard, Kalong (KEP) in 1929 (FMS 24048) from Ulu Kelau, Pahang. The specimen consists of two detached leaves and a detached inflorescence mounted on one sheet. Its vernacular name, *Akar telinga berok* (the pig-tail macaque’s ear climber in Malay) indicates that it is a climber. After a lag of 70 years, Kiew collected a flowering specimen (RK 4879) in the Awana waterfall area, Genting Highlands, Pahang. The specimen is complemented by good field notes and was identified as *Aristolochia* sp.

Recently, I was asked to identify a leaf (Kiew s.n., barcode KEP196081) of a butterfly larva food plant collected in the Genting Tea Estate, Pahang. This instigated me to make a visit to the estate, which revealed that the plant is conspecific with the two specimens mentioned above. According to H.S. Barlow and S.K.L. Hok (*pers*. *comm*.), larvae of the butterfly species, *Parides (Atrophaneura) sycorax egertoni* (Distant) a member of the family Papilionidae, commonly known as the White Head Batwing (Malay name: *Kepala Putih*) feed on the leaves of this species. Their observations in the Genting Tea Estate revealed that its larvaedefoliate young plants and then girdle the stem base just before they metamorphose into pupae. The plant manages to re-sprout later.

## Taxonomy

### Aristolochia vallisicola

T.L.Yao sp. nov.

urn:lsid:ipni.org:names:77120982-1

http://species-id.net/wiki/Aristolochia_vallisicola

**Figure 1.**
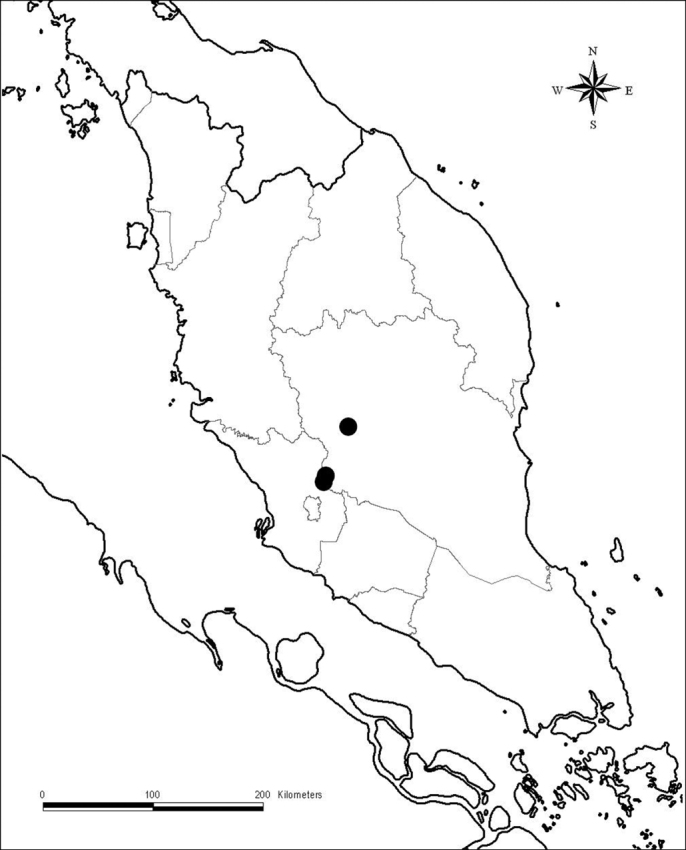
Distribution of *Aristolochia vallisicola* (●).

Figures 1 [Fig F2] 3

#### Note.

This species differs from all other Peninsular Malaysian *Aristolochia* L. species in its lamina with pinnate lateral veins, inflorescence with a long peduncle, its disc-shaped perianth limb, annulated hairy perianth mouth and 3-lobed gynostemium. This species is similar to *Aristolochia coadunata* Backer in the lanceolate or oblanceolate lamina with pinnate lateral veins but differs in its larger disc-shaped perianth limb, 58–65 mm diam. versus 15–30 mm diam. in *Aristolochia coadunata* and its longer peduncle, 15.5–17 cm long versus up to 2 cm long in *Aristolochia coadunata*. This species is also similar to *Aristolochia versicolor* S.M.Huang in the lanceolate or oblanceolate lamina with pinnate lateral veins but differs in its longer petiole, 2.5–7 cm long versus 1–2 cm long, broader leaves, at least 7.5 cm wide versus to 6.5 cm wide, and longer peduncle, 15.5–17 cm long versus 2–3(–10) cm long in *Aristolochia versicolor*. The summary and other characters comparison is presented in Table 1.

**Type.** **Peninsular Malaysia**. Pahang: Genting Highlands, Awana Waterfall. 26 November 1999 (fl), R.Kiew 4879 (holotype SING!, barcode 78162).

**Table 1.** Comparison of *Aristolochia vallisicola*, *Aristolochia coadunata* and *Aristolochia versicolor*.

Characters	*Aristolochia vallisicola*	*Aristolochia coadunata	**Aristolochia versicolor (China)	***Aristolochia versicolor (Thailand)
Petiole length; indumentum	2.5–7 cm long; puberulous	3–9 cm long; pubescent	1–2 cm long; sparsely pilose	1–2 cm long; adpressed hairy
Lamina; length by width (cm)	lanceolate, oblanceolate or broadly oblanceolate; 15–24 × 7.5–14	ovate oblong to lanceolate, rarely ovate; 7.5–33 × 4–12	narrowly elliptic to lanceolate-elliptic; 14–25 × 4–6.5	oblanceolate, oblong-oblanceolate, or elliptic oblong; 11.2–17.5 × 3.4–4.7
Lamina base; sinus depth (mm)	cordate; 2–3	cordate; 5–10	narrowly auriculate; 5–7	narrowly, slightly cordate
Pinnate lateral vein pairs	6–7	4–6	9–10	7–8
Inflorescence	cauline; peduncle 15.5–17 cm long, divided into 4–5 internodes of different lengths	in axils of foliage leaves, rarely cauline; peduncle up to 2 cm long	in axils of foliage leaves, peduncle 2–3 cm long	in axils of foliage leaves, peduncle ca 10 cm long
Bract indumentum	pilose	puberulous	—	pilose
Perianth	tube geniculately curved, utricle cylindric, ca 30 × 8 mm, tube ca 35 × 8 mm; limb disc-shaped, 58–65 mm diam., 3-lobed, mouth annulate	tube geniculately curved, utricle ovoid tubular, 35–30 × 7 mm, tube cylindric, 30–45 × 6 mm, limb disc-shaped, 15–30 mm diam., obscurely 3-lobed, mouth not annulate	tube geniculately curved, basal portion of tube 30–40 × 6–8 mm; limb disc-shaped, 40–60 mm diam., 3-lobed, mouth annulate	tube geniculately curved, utricle ovoid, 8–10 × 8–12 mm, tube ca 13–23 × 5–7 mm; limb disc-shaped, 46–50 mm diam.
**Distribution**	Peninsular Malaysia	Sumatra, Java, ^†^Peninsular Malaysia (Hou 1984; Igarashi and Fukuda 1999)	China: Guangdong, Guangxi, Yunnan (Huang et al. 2003)	North Eastern Thailand (Phuphathanaphong 1987)

* Images of Backer 26130 (L), Bosscha s.n.(BO-108722) (BO), Schouten s.n. (BO-108723 & BO-108735) (BO),van Steenis 4317, 7326, 12625 (L) seen. Comparison also based on species description and drawings (Backer 1920; Hou 1984, fig. 12; van Steenis 2006, colour plate 4).

** Type specimen could not be located. Comparison based on species description and line drawings (Hwang 1981, fig. 4; Huang et al. 2003, fig. 222, 4–6).

*** Comparison based on images of Beusekom and Phengklai 2985 (L) and its line drawing, and species description (Phuphathanaphong 1987, fig. 13).

^†^ Igarashi and Fukuda (1997) recognised *Aristolochia coadunata* as occurring in Peninsular Malaysia and mentioned that it is one of the food plants of *Parides (Atrophaneura) sycorax*. I have not seen any *Aristolochia coadunata* specimens from Peninsular Malaysia.

#### Description.

Slender climber. Stem ca 2.5 mm thick, surface shallowly furrowed, sometimes smooth, puberulent, trichomes hooked. **Leaves:** petiole twisted, 2.5–7 cm long, ca 2.5 mm thick, puberulent, indumentum a mix of hooked and straight hairs; lamina lanceolate or narrowly oblanceolate or oblanceolate, 15–24 × 7.5–14 cm; base cordate, auricles rounded, sinus 2–3 mm deep, 8–12 mm wide, margin entire, apex acute; leathery; lamina surface above glabrescent, with scattered black gland dots, lamina surface below puberulent, indumentum a mix of longer straight and shorter hooked hairs; midrib above sunken, below prominent; lateral veins pinnate, above faint, below prominent, basal pair 1, pinnate pairs 5–7; intercostal veins net-like. **Inflorescences** cauline, solitary; peduncle branched once; 15.5–17 cm long, ca 2 mm thick, puberulent, indumentum mainly of hooked trichomes, scattered with long spreading hairs. Bracts ovate, ca 3 × 1.5 mm, pubescence, base cuneate, apex acute. **Flowers:** pedicel ca 40 mm long, ovary ca 13 × 2 mm, villous; perianth glossy, greyish pale orange with purple tinge, purple beneath, ca 6.5 cm long, outer surface sparsely villose with shorter hooked trichomes, tube geniculately curved, utricle cylindric, ca 30 × 8 mm, inner surface with a glistening white patch of stellate trichomes, perianth tube ca 35 × 8 mm, limb disc-shaped, 5.8–6.5 cm diam., 3-lobed, venation faint, mouth annulate, villous; gynostemium in transverse section faintly trigonal; stamens 6, anthers ca 3 × 0.3 mm; stigmatic lobes 3, conical, ca 0.8 mm long, apex blunt. **Fruit** and **seed** unknown.

**Figure 2. F2:**
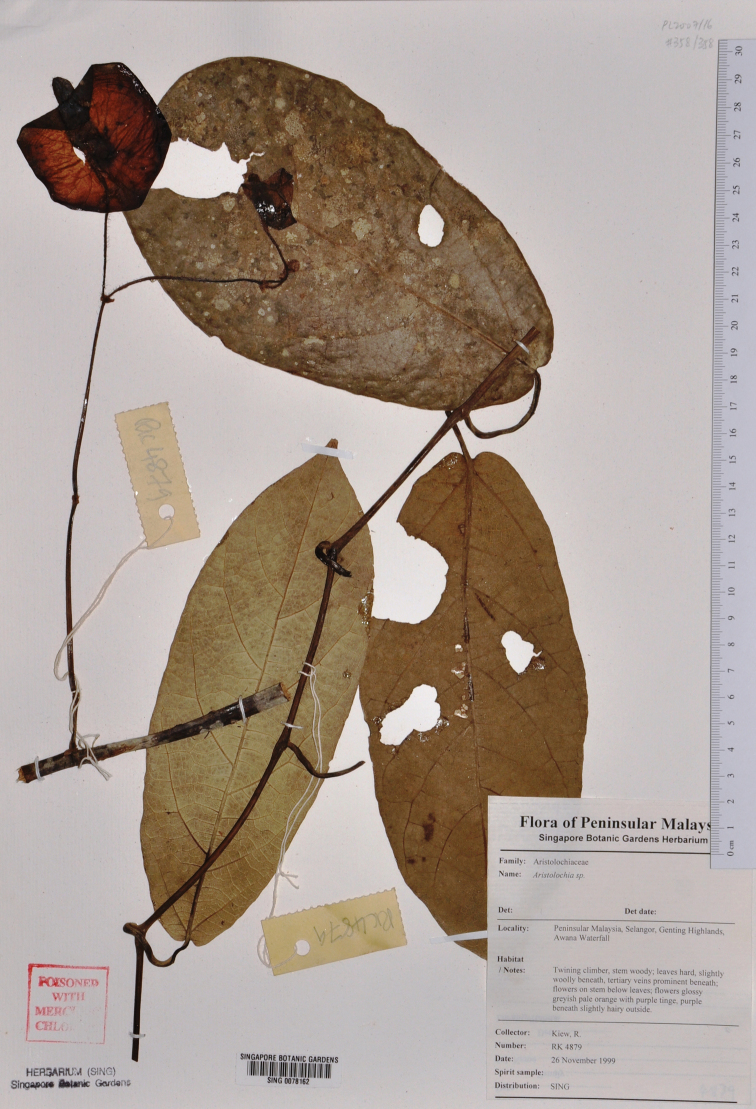
Type specimen of *Aristolochia vallisicola* (Kiew RK 4879, SING, barcode 78162).

**Figure 3.**
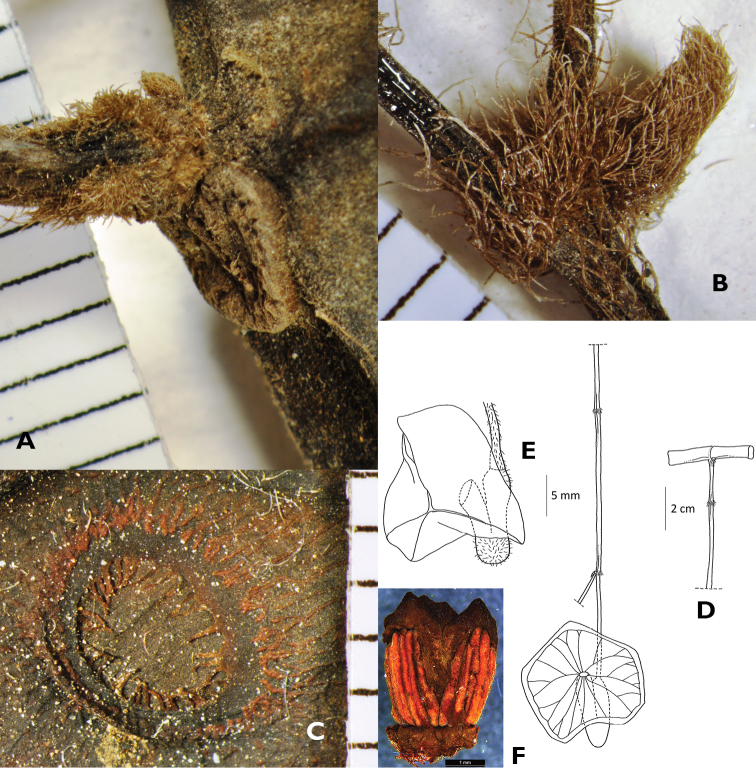
*Aristolochia vallisicola* T.L. Yao, **A** insertion of an inflorescence in axil of petiole scar at thickened stem node **B** villous inflorescence bract **C** annulated perianth mouth **D** an inflorescence with an opened flower **E** flower bud **F** gynostemium. (All from Kiew RK 4879.)

#### Vernacular name.

*Akar telinga berok* (Malay).

#### Distribution.

*Aristolochia vallisicola* isendemic in Peninsular Malaysia, Pahang. It has only been found on Titiwangsa Range and its vicinities.

#### Ecology.

Thisspecies occurs in highland valleys of lower montane forest about 1000 m altitude and often by rocky streamsides. Specimens with flowers werecollected in September and November.

#### Etymology.

The species name *vallisicola* denotes its habitat preference for valleys.

#### Conservation status.

Least Concern. This species occurs above 1000 m altitude, a habitat which is protected by Malaysian legislation.

#### Specimens examined.

Peninsular Malaysia, Pahang: Ulu Kelau, Raub, 24 September 1929 (fl), Kalong FMS 20248 (KEP, barcode 196080); Genting Tea Estate, R. Kiew s.n. (KEP, barcode 196081).

## Discussion and conclusion

*Aristolochia vallisicola* with disk-shaped perianth of 3 lobes which valvate in bud, annulated perianth mouth and gynostemium with 3 segments each consisting 2 stamens belongs to *Isotrema* (Huber 1993). *Isotrema* consists of ca 50 species distributed in temperate and tropical East Asia and in North and Central America. The new species presented here is its first record in Peninsular Malaysia. The position of *Isotrema* clade within *Aristolochia* *s.l*. is confirmed by phylogenetic studies (Kelly and González 2003; Ohi-Toma et al. 2006).

Old World *Aristolochia* species with a disc-shaped perianth limb are common in northern India (Hooker 1886; Karthikeyan et al. 2010) and southern China (Huang et al. 2003) while 1-lipped or 3-lobed perianth limb are prevalent in Malesian *Aristolochia* species (Hou 1984). *Aristolochia vallisicola* is the only species with a disc-liked perianth limb in Peninsular Malaysia. Apparently, it is a link between the Asian Continental element and Sumatran-Javanese *Aristolochia coadunata*.

Speciesof *Aristolochia*, a genus of high climber or woody lianas in Malesian forests, are not easy sighted and are very often represented by meagre herbarium specimens. Furthermore, the plants are rarely found in flower. In the past 15 years, 8 new species of *Aristolochia* were described from Thailand (González and Poncy 1999; Hansen and Phuphatanaphong 1999; Phuphathanaphong 2006). This indicates that the species diversity of *Aristolochia* in the Old World, especially in South East Asia is still underestimated. I predict more novelties will be discovered when more specimens from this region are available for taxonomic study.

## Supplementary Material

XML Treatment for Aristolochia vallisicola
